# Association of GATA3, P53, Ki67 status and vascular peritumoral invasion are strongly prognostic in luminal breast cancer

**DOI:** 10.1186/bcr2249

**Published:** 2009-04-30

**Authors:** Jocelyne Jacquemier, Emmanuelle Charafe-Jauffret, Florence Monville, Benjamin Esterni, Jean Marc Extra, Gilles Houvenaeghel, Luc Xerri, François Bertucci, Daniel Birnbaum

**Affiliations:** 1 Département d'Oncologie Moléculaire Centre de Recherche en Cancérologie de Marseille, Institut Paoli-Calmettes, UMR891 Inserm; IFR137, 232 Bd sainte Marguerite Marseille, 13009 France; 2 Département de BioPathologie, Institut Paoli-Calmettes, 232 Bd sainte Marguerite Marseille,13009 France; 3 UFR de Médecine, Université de la Méditerranée, 58 Bd Charles Livon Marseille 13284, France; 4 Département de Biostatistiques, Institut Paoli-Calmettes, 232 Bd Ste Marguerite Marseille 13009, France; 5 Département d'Oncologie Médicale, Institut Paoli-Calmettes, 232 Bd Ste Marguerite Marseille 13009, France; 6 Département d'Oncologie Chirurgicale, Institut Paoli-Calmettes, 232 Bd Ste Marguerite Marseille 13009, France

## Abstract

**Introduction:**

Breast cancers are traditionally divided into hormone-receptor positive and negative cases. This classification helps to guide patient management. However, a subgroup of hormone-receptor positive patients relapse irrespective of hormonal therapy. Gene expression profiling has classified breast tumours into five major subtypes with significant different outcome. The two luminal subtypes, A and B, show high expression of *ESR1*, *GATA3 *and *FOXA1 *genes. Prognostic biomarkers for oestrogen receptor (ER)-positive cases include progesterone receptor (PR) and androgen receptor (AR), and proteins related to proliferation or apoptotic resistance. The aim of this study was to identify the best predictors of success of hormonal therapy.

**Methods:**

By immunohistochemistry we studied 10 markers in a consecutive series of 832 cases of breast carcinoma treated at the Paoli-Calmettes Institute from 1990 to 2002 and deposited onto tissue microarrays (TMA). These markers were luminal-related markers ER, PR, AR, FOXA1 and GATA3 transcription factors, proliferation-related Ki67 and CCND1, ERBB2, anti-apoptotic BCL2 and P53. We also measured vascular peritumoural invasion (VPI), size, grade and lymph node involvement. For 143 cases, gene expression profiles were available. Adjuvant chemotherapy and hormonal therapy were given to high- and low-risk patients, respectively. The 162 events observed and taken into account were metastases.

**Results:**

Molecular expression of the 10 parameters and subtype with ER status were strongly correlated. Of the 67 luminal A cases of this series, 63 were ER-positive. Multivariate analyses showed the highly significant prognostic value of VPI (hazard ratio (HR) = 2.47), Ki67 (HR = 2.9), P53 (HR = 2.9) and GATA3 (HR = 0.5) for the 240 patients who received hormonal therapy.

**Conclusions:**

A panel of three antibodies (Ki67, P53 and GATA3) associated with VPI can significantly improve the traditional prognosticators in predicting outcome for ER-positive breast cancer patients receiving hormonal therapy.

## Introduction

The traditional division of breast cancers into hormone receptor positive and negative cases helps to guide patient management. However, a subgroup of hormone receptor-positive patients relapse irrespective of standard hormonal therapy. Gene expression profiling has classified breast tumours into five major molecular subtypes with different outcomes. The two luminal subtypes, A and B, express the *ESR1*, *GATA3 *and *FOXA1 *genes [1].

Compared with luminal A, luminal B tumours have a poor prognosis [1-3]. However, there are few indicators to determine if the response to hormonal therapy is different between A and B subtypes. In a previous study we validated a non-linear algorithm including six immunohistochemical markers on tissue microarrays (TMA): oestrogen receptor (ER), progesterone receptor (PR), ERBB2, BCL2, P53 and MYC [4]. This algorithm had strong prognostic value in ER-positive patients with or without hormonal therapy. The difference between luminal A and B was not investigated in this study. In another study we showed that the subset of patients with luminal A tumours, called Ab, which express mitotic kinases had a poorer prognosis than the majority that do not express these kinases [5]. This subset with high kinase score had a prognosis close to luminal B tumours. In fact, luminal Ab resemble luminal B tumours; they are distinguished only because the lists of genes used in gene expression analyses to identify subtypes are not accurate enough and because luminality reflects a continuum from poorly differentiated, highly proliferative (luminal B) to well-differentiated, poorly proliferative (luminal Aa).

The prognostic distinction between luminal Aa and Ab suggest that grade and P53 are also involved but the kinase score was associated with the highest hazard ratio (HR). In the absence of reliable antibodies the kinase score is difficult to implement in a routine setting. We therefore searched for easily identifiable factors that could be associated with the prognosis of patients receiving hormonal therapy for the different luminal subtypes.

P53 mutation is generally associated with basal breast cancer. However, we demonstrated its impact in luminal cases [5]. P53 expression observed in BRCA1 luminal cases correspond to a true mutation in only four of seven cases [6]. This suggests that P53 expression could be associated with proliferation in luminal cases independent of mutation.

Quantitative ER status is correlated with a strong response to hormonal therapy. PR, GATA3 and FOXA1, and proteins related to proliferation or apoptotic resistance such as BCL2 could also influence hormonal response. The transcription factor GATA3 is a defining marker of the luminal subtypes. GATA3 has an essential role in the morphogenesis of the mammary gland and actively maintains luminal epithelial differentiation [7]. We demonstrated a good correlation between GATA3 gene and protein expression [2]. A recent meta-analysis [7] showed that both ER-alpha and GATA3 are coexpressed with ER-alpha-associated genes such as PS2/TFF1, TFF3, FOXA1, BCL2, ERBB4, XBP1, NRIP, IL6ST, Keratin 18 and cyclin D1/CCND1. The transcription factor FOXA1 is a downstream target of GATA3 in the mammary gland. FOXA1 expression is associated with that of ER, PR and androgen receptor (AR) [8-10] and with a better survival. FOXA1 binds to chromatinised DNA, opens the chromatin and enhances binding of ER-alpha. Thus, a network comprising GATA3, FOXA1, ER-alpha and oestrogen constitutes a major proliferation and survival signal for luminal A breast cancer [11].

Many human breast cancers express AR. A recent study of AR on formalin-fixed, paraffin-embedded archival specimens of 200 cases of breast cancer showed that 60% of invasive carcinoma and 82% of ductal carcinoma *in situ *were AR-positive [12]. The great majority of well-differentiated carcinomas were both AR and ER-positive. In contrast, 39% of poorly-differentiated carcinomas were ER-negative but AR-positive. The clinical value of AR expression is unclear. However, AR expression was strongly correlated with ER in a series of 842 breast carcinomas [13]. Few studies suggest the impact of AR on the response to hormonal therapy [14].

Finally, a recent meta–analysis confirmed that BCL2 has an independent prognostic impact [15]. However, no prospective study has shown the predictive impact of BCL2 expression in ER-positive cases.

The aim of our study was to identify the prognosis of patients receiving hormonal therapy among histoclinical and immunohistochemical factors.

## Materials and methods

### Patients

We studied a consecutive series of 832 tumours with early (stage I, II or III) breast cancer treated in our institution between October 1987 and December 2001 and with sufficient cancer tissue available for inclusion in TMA. The stage of disease was defined according to the tumour node metastasis (TNM) classification. Tumours were all invasive adenocarcinomas. The patients were treated according to guidelines used in our institution: all had primary surgery that included complete resection of the tumour (modified radical mastectomy in 28% of cases, lumpectomy in 72%) and axillary lymph node dissection; 96% were treated with breast-conservative surgery received adjuvant local-regional radiotherapy; 51.3% were given adjuvant chemotherapy (anthracyclin-based regimen in most cases); 56.5% received adjuvant hormone treatment (tamoxifen in most cases) and 54.9% of these received adjuvant chemotherapy. After completion of treatment, the patients were evaluated at least twice a year for the first five years and at least annually thereafter. The median follow-up was 86 months after diagnosis; 162 patients experienced metastatic relapse as a first event (local recurrence was not taken into account as first event). The five-year metastasis-free survival (MFS) rate was 83.2% (95% confidence interval (CI) = 80.4 to 85.8). The experimental part of this study concerning paraffin-embedded samples was completed before informed consent was necessary but was approved and executed in compliance with our institutional review board. Each sample was assigned an anonymous unique identification that was linked to an anonymous clinical board approved data base containing follow-up information. The study was performed with the intent of benefiting treatment planning in future patients.

### Breast cancer samples

Tissues were collected from 143 patients with invasive adenocarcinoma who underwent initial surgery at the Institut Paoli-Calmettes (Marseilles, France). Each patient gave written informed consent. Samples were macro-dissected and frozen in liquid nitrogen within 30 minutes of removal.

### DNA and RNA extraction

Nucleic acids were extracted from frozen samples by using guanidium isothiocyanate and cesium chloride gradient, as previously described [16]. RNA integrity was controlled on the Agilent Bioanalyzer (Agilent Technologies, Massy, France).

### Gene expression profiling with DNA microarrays

Gene expression was analysed in 143 breast cancer samples and four normal breast samples with Affymetrix U133 Plus 2.0 human oligonucleotide microarrays (Affymetrix Santa Clara, CA, USA). Preparation of c-RNA, hybridisations, washes and detection were performed as recommended by the supplier. For each sample, synthesis of the first-strand c-DNA was done from 3 μg total RNA by T7-oligo(dT) priming, followed by second-strand cDNA synthesis. After purification, *in vitro *transcription associated with amplification generated cRNA-containing biotinylated pseudouridine. Biotinylated cRNA was purified, quantified and chemically fragmented (95°C for 35 minutes), then hybridised to microarrays in 200 μL hybridisation buffer at 45°C for 16 hours. Automated washes and staining with streptavidin-phycoerythrin were performed as recommended. Double signal amplification was achieved by biotinylated antistreptavidin antibody with goat-IgG blocking antibody. Scanning was performed with Affymetrix GeneArray scanner and quantification with Affymetrix GCOS software.

### Gene expression data analysis

Affymetrix data were analysed by the Robust Multichip Average method in R using Bioconductor and associated packages [17]. The Robust Multichip Average performed background adjustment, quantile normalisation and summarisation of 11 oligonucleotides per gene. Before analysis, a filtering process removed the genes with low and poorly measured expression, as defined by an expression value inferior to 100 units in all breast cancer tissue and normal tissue samples, from the dataset. All data was then log_2_-transformed for display and analysis.

Basal and luminal breast cancers were distinguished by the differential expression of clusters of genes. Sub-classification of the luminal cases was done as previously described [5]. Kinase gene expression identified two subgroups of luminal A breast cancers, that is luminal Aa and Ab.

### Tissue microarrays construction and immunohistochemistry

TMAs were prepared as previously described [18] from formalin-fixed and paraffin-embedded tissue. For each tumour, three representative areas were selected from a H&E-safran-stained section of a donor block. Core cylinders with a diameter of 0.6 mm each were punched from each of these areas and deposited into three separate recipient paraffin blocks using a specific arraying device (Alphelys, Plaisir, France).

Immunohistochemistry of 5 μmm TMA sections was performed as previously described using Dako LSAB^R^2 Kit in the autoimmunostainer (Dako Autostainer, Glostrup, Denmark). Sections were deparaffinised in Histolemon (Carlo Erba Reagenti, Rodano, Italy) and rehydrated in graded ethanol solutions. Results were evaluated under a light microscope by two pathologists (EC-J, JJ) and scored by the quick score (QS) [19]. The QS was used to combine the impact of the percentage and the intensity of the immunostaining. QS multiplies the percentage by the intensity and represents a range of 0 to 300. For each antibody, a sample was considered as positive when the QS was strictly superior to 0. However, the Ki67 status was expressed in terms of percentage of positive cells, with a threshold of 20% of positive cells. The ERBB2 status was evaluated with the Dako scale (HercepTest kit scoring guidelines, DakoCytomation, Copenhagen, Denmark). The level of 3+ was considered as positive and all 2+ cases were evaluated by chromogen in situ hybridisation (only the case with a ratio higher than 2.2 were considered as positive).

For each tumour, the mean of the score of a minimum of two core biopsies was calculated. The list of antibodies used is given in Table 1.

**Table 1:** Immunohistochemical antibodies used to characterize the luminal cases

**Protein (Clone)**	**Antibody**	**Origin**	**Clone**	**Pre-treatment**	**Dilution**	**Location of staining**	**Normal**
**Androgen receptor** **(AR)**	mmb	Dako	4AR441	PH9 Target retrieval solution (98°C, 40 min)	1/50°	nucleus	+
**BCL2**	mmb	Dako	124	Citrate*	1/100°	cytoplasm	+
**CyclinD1** **CCND1**	mmb	Labvision	SP4	EDTA*	prediluted	nucleus	+
**ERBB2**	mmb	Dako Herceptest Ltd	AO485	Target retrieval *	1/500	membrane	-
**Estrogen receptor (ER)**	mmb	Novocastra laboratories Ltd	6F11.2	Target retrieval *	1/60	nucleus	+
**FOXA1**	mmb	AbNova	2D7	Citrate*	1/250	nucleus	+
**GATA3**	mmb	Santacruz	HG3-31	Citrate*	1/100°	nucleus	+
**P53**	mmb	Beckmann	DO-1	Citrate*	1/4	nucleus	-
**Progesterone** **receptor (PR)**	mmb	Dako	PFR 636	Target retrival *	1/80	nucleus	+
**Ki67**	mmb	Dako	KI-67	Target retrieval solution *	1/100	nuclear	+

+ = positive expression, - = negative expression.

The asterisk symbols mean that it is a buffer solution.

### Statistical analysis

Survival rates were estimated by using the Kaplan-Meier method [20]. The endpoint was the MFS, which was defined as the time from the date of breast cancer diagnosis until the date of the first distant relapse. Patients without relapse were censored at the time of last follow-up. Survival analysis was computed with a stratification on treatment by chemotherapy. Relative risks of metastasis according to the baseline factors were estimated by using the Cox proportional-hazards regression models [21] in univariate and multivariate analyses. In univariate analysis, differences in MFS were analysed by the Log-Rank test. Factors with a *P *value less than 0.15 in univariate analysis were included in the multivariate analysis, with a backward selection of variable procedure to minimise the Akaike information criterion [22]. Results are presented as mean (95% CI). Statistical analyses were performed with the R.2.7.1. Statistical language [23].

## Results

### Correlation between molecular subtype and oestrogen receptor immunohistochemical status

A total of 135 of the 143 cases showed good-quality RNA and profiles. The correlation between expression of each parameter between microarrays and QS was excellent and highly significant (Table 2). The lowest level of the Rho coefficient was observed for Ki67, P53 and ERBB2.

**Table 2:** Correlation between expression in microarray and quick score of the ten markers analysed

Spearman correlation test	AR	BCL2	CCND1	ERBB2	FOXA1	GATA3	KI67	P53	ER	PR
Rho *	0.49	0.56	0.43	0.28	0.58	0.64	0.32	0.30	0.73	0.69
P	< 0.0001	< 0.0001	< 0.0001	0.00067	< 0.0001	< 0.0001	0.00018	0.00023	< 0.0001	< 0.0001

Rho test the non nullity of the correlation (Spearman test). The level of signification is expressed by the *P* value.

The frequency of the different subtypes was: 25% basal, 12% ERBB2, 46% luminal A (68.1%% Aa and 31.8%% Ab), 2% luminal B and 9% normal-like. Molecular subtype and ER status were strongly correlated. Only 8% of basal breast cancers were ER-positive for 23.5% of ERBB2, 95.5% of luminal A, 100% of luminal B and 53.8% of normal-like breast cancers (Table 3).

**Table 3:** Correlation between molecular subtype and immohistochemical ER status

Subtype N = 135	Basal N = 36 (27%)	ERBB2 N = 17 (12.5%)	LuminalAa N = 45 (33%)	LuminalAb N = 21 (15.5%)	LuminalB N = 3 (2%)	Normal-like N = 13 (10%)
ER-positive N = 81	3 (8%)	4 (23.5%)	43 (95.5%)	21 (100%)	3 (100%)	7 (53.8%)
ER-negative N = 54	33 (91.6%)	13 (76.4%)	2 (4.5%)	0	0	6 (46.2%)
*P*-value	4.5910^-13^	0.0029	9.6 10^-11^	4.9 10^-6^	NS	NS

### Univariate and multivariate analyses of survival

We studied the impact of 16 histoclinical and immunohistochemical factors on disease-free survival. Hormonal therapy, size of the lesion, histoprognostic grade, vascular peritumoural invasion (VPI), ER, BCL2, GATA3, Ki67 and P53 had significant impact (Table 4). Only age, CCND1, PR, FOXA1 did not have any significant value in MFS.

**Table 4:** Univariate analysis of 832 consecutive cases of breast carcinomas and 16 factors including classical histopronostic and immunohistochemical parameters

**Variable**	**NE (%)**	**Classes**	**N (%)**	**Hazard Ratio [IC95]**	**p-value**
**Hormonal therapy**	0 (0%)	no	361 (43%)	1	0.000209
		yes	471 (57%)	0.558 [0.408–0.763]	

**Age**	1 (0%)	< 45 years	104 (13%)	1	0.673
		>= 45 years	727 (87%)	1.1 [0.711–1.696]	
		< 45	104 (13%)	1	0.145

**Tumor Size**	7 (1%)	< 25 mm	381 (46%)	1	< 0.0001
		>= 25 mm	444 (54%)	2.47 [1.719–3.562]	

**Scarff-Bloom-Richardson grade**	22 (3%)	I	266 (33%)	1	< 0.0001
		II–III	544 (67%)	3.69 [2.244–6.083]	

**VPI**	4 (0%)	Absent	536 (65%)	1	< 0.0001
		present	292 (35%)	2.1 [1.519–2.897]	

**Lymph node invasion**	16 (2%)	N-	442 (54%)	1	< 0.0001
		N+	374 (46%)	2.61 [1.693–4.013]	

**AR**	175 (21%)	0	220 (33%)	1	0.0122
		> 0	437 (67%)	0.65 [0.463–0.913]	
		< 80	410 (62%)	1	0.00422
		>= 80	247 (38%)	0.568 [0.383–0.841]	

**BCL2**	201 (24%)	0	162 (26%)	1	0.000944
		> 0	469 (74%)	0.548 [0.381–0.787]	

**CCND1**	142 (17%)	0	284 (41%)	1	0.562
		> 0	406 (59%)	0.905 [0.647–1.268]	

**ERBB2**	123 (15%)	0 or 1	621 (88%)	1	0.209
		2 or 3	88 (12%)	1.33 [0.852–2.071]	

**FOXA1**	187 (22%)	0	40 (6%)	1	0.324
		> 0	605 (94%)	1.43 [0.698–2.952]	

**GATA3**	188 (23%)	0	247 (38%)	1	0.00024
		> 0	397 (62%)	0.534 [0.38–0.75]	

**Ki67**	171 (21%)	< 20	560 (85%)	1	0.000748
		>= 20	101 (15%)	1.96 [1.316–2.923]	

**P53**	140 (17%)	0	525 (76%)	1	0.00095
		> 0	167 (24%)	1.76 [1.253–2.47]	

**ER**	79 (9%)	0	169 (22%)	1	0.000276
		> 0	584 (78%)	0.54 [0.386–0.756]	

**PR**	120 (14%)	0	263 (37%)	1	0.0875
		> 0	449 (63%)	0.752 [0.542–1.044]	

NE: Number of non evaluable or miss cases on tissu microarrays.

For ERBB2 there was a significant difference in terms of disease-free survival at 60 months with 83.7% for the negative cases and 69.1% for the 3+ cases and amplified 2+ (*P *= 0.017). However, when the analysis was stratified on the presence or not of chemotherapy, no significant difference between the two groups was noted.

Eight factors were retained by the multivariate analysis including histopronostic grade, axillary lymph node invasion, VPI, size, then Ki67, P53, BCL2 and hormonal therapy (Table 5).

**Table 5:** Significant parameters retained by Cox multivariate analysis in the estrogen receptor positive cases

**N = 378**		**Coefficient**	**HR**	**IC95**	**p-value**
**Hormonal therapy**	no		1		
	yes	-0.897	0.408	[0.257–0.647]	0.00014

**Size**	< 25 mm		1		
	>= 25 mm	0.9	2.46	[1.449–4.175]	0.00085

**Scarff-Bloom-Richardson grade**	I		1		
	II–III	0.521	1.68	[0.805–3.522]	0.17

**VPI**	no		1		
	yes	0.657	1.93	[1.213–3.068]	0.0055

**Lymph node**	N-		1		
	N+	0.68	1.97	[1.208–3.222]	0.0066

**BCL2**	< 160		1		
	>= 160	-0.613	0.542	[0.331–0.886]	0.015

**Ki67**	< 20		1		
	>= 20	0.641	1.9	[1.167–3.086]	0.0098

**P53**	0		1		
	> 0	0.369	1.45	[0.928–2.254]	0.1

### Molecular subtype and oestrogen receptor positivity

In the restricted ER-positive population studied by gene expression profiling we observed that 67 of 81 (86.4%) were luminal cases (Table 6). There was no difference in ER-positivity level (with a cut off QS of 120) between luminal Aa (18 of 43 above 120, 41.8%) and luminal Ab (8 of 21 above 120, 38%) cases. However, a significant difference was observed for proliferation: luminal Ab showed a higher grade (*P *= 4.710; Table 6) and a higher Ki67 index (*P *= 0.02) than luminal Aa. The three luminal B were ER-positive (the percentage of ER-positive cells was below 5% for two cases) but grade 3. Two were positive for P53.

**Table 6:** Correlation between the different subtypes and histopronostic and immunohistochemistry factors in ER-positive cases

	Basal	ERBB2	Luminal Aa	Luminal Ab	Luminal B	Normal-like	p-value
ER-positive N = 84	3	4	43 (%)^x^	21(%)^x^	3	7	3 cases non evaluable
Grade I	1	0	**14(32.5%)**	**0**	0	5	**4.710**^-6^
Grade II	0	0	**24(55.8%)**	**7(30%)**	0	2	
Grade III	2	4	**5(11.6%)**	**14(66.6%)**	3	0	
VPI positive	0	1	20/42 (47.6%)	10/21 (47.6%)	3	1	
Positive lymph nodes	1	2	26/43 (60.4%)	15/21 (71.4%)	2	2	
AR positive	1	2	31/36(86%)	15/21(71.4%)	2	3	
BCL2 positive	2	2	36/39 (92.3%)	14/15 (93%)	2	6/6 (100%)	
CCND1 Positive	2	0	26/41 (63,4%)	13/19 (68%)	2	2/5	
ERBB2 (2+/3+)	0	3 (3+)	3/43 (6.9%)	0	1(2+)	0	
FOX A1 positive	1	3	38/39 (97.4%)	19/19 (100%	3	4/5	
GATA3 Positive	0	1	29/36 (80.5%)	12/18 (66.6%)	3	4/5	
Ki67 > 20%	1	0	**2/40(5%)**	**5/18(27.7%)**	0	0	**0.02**
P53 positive	2	2	8/42 (19%)	4/20 (20%)	1	0/6	
PR positive	0	0	37/43 (86%)	16/20 (80%)	2	5/6	

Numbers in bold mean the percentage of positive available cases.

One-third of the luminal A were Ab (31.8%) and two-thirds (68.1%) were luminal Aa cases. The four ERBB2-subtype cases were ER-positive but the level of ER expression was lower than the median value of QS 120; all the cases were grade 3 and PR-negative. The seven normal-like cases were grade 1 (n = 5) or 2 (n = 2) and four showed a low level of ER protein expression.

### Markers and survival

**Figure 1:**
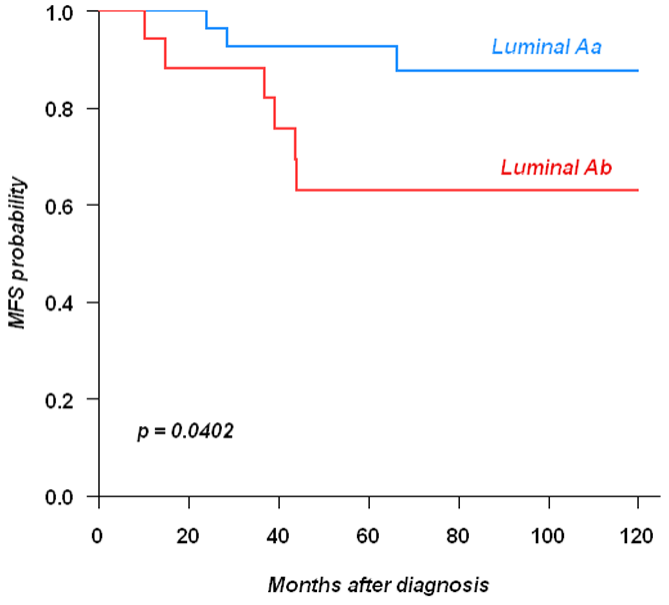
Influence of the molecular subtype on metastasis-free survival in ER-positive cases receiving hormonal therapy. Log-Rank test: *P *= 0.0402. ER = oestrogen receptor; MFS = metastasis-free survival.

We then restricted the study to the ER-positive cases treated by hormonal therapy (n = 384). Subtype status was available for only a small series of these cases (n = 43). MFS was different between luminal Aa and Ab cases (*P *= 0.042; Figure 1). Of the 14 factors studied in univariate analysis (Table 7) only 6 showed a different distribution: size, grade, VPI, lymph node invasion, GATA3 and Ki67. Oestrogen-related proteins such as FOXA1 and AR had no significant impact whatever their quantitative value. The ER and PR level of expression had no significant MFS value in univariate analysis. The multivariate analysis in terms of MFS retained four factors: VPI, Ki67, P53 and GATA3 (Table 8).

**Table 7:** Univariate analysis of ER-positive cases with adjuvant hormonal

**Variable**	**NE (%)**	**Classes**	**N (%)**	**Hazard Ratio [IC95]**	**p-value ×**
**Age**	0 (0%)	< 45 years	29 (8%)	1	0.964
		>= 45 years	355 (92%)	1.02 [0.401–2.605]	
		< 45	29 (8%)	1	0.315
		45–60	155 (40%)	0.834 [0.312–2.226]	
		> 60	200 (52%)	1.32 [0.493–3.522]	

**Tumoral size**	2 (1%)	< 25 mm	141 (37%)	1	0.0222
		>= 25 mm	241 (63%)	2.16 [1.1–4.23]	

**Scarff-Bloom-Richardson grade**	2 (1%)	I	141 (37%)	1	0.000487
		II–III	241 (63%)	4.1 [1.74–9.644]	

**VPI**	3 (1%)	absent	237 (62%)	1	0.00363
		present	144 (38%)	2.36 [1.305–4.268]	

**N**	3 (1%)	N-	174 (46%)	1	0.0495
		N+	207 (54%)	2.22 [1–4.921]	
		N < 3	288 (76%)	1	< 0.0001
		N >= 3	93 (24%)	3.81 [2.113–6.883]	

**AR**	66 (17%)	0	74 (23%)	1	0.306
		> 0	244 (77%)	0.712 [0.371–1.369]	

**BCL2**	66 (17%)	0	38 (12%)	1	0.0676
		> 0	280 (88%)	0.492 [0.226–1.069]	

**CCND1**	47 (12%)	0	97 (29%)	1	0.971
		> 0	240 (71%)	1.01 [0.542–1.886]	

**ERBB2**	36 (9%)	1 or 2	319 (92%)	1	0.558
		2 or 3	29 (8%)	1.32 [0.519–3.359]	

**FOXA1**	78 (20%)	0	3 (1%)	1	0.406
		> 0	303 (99%)	9220000 [0-Inf]	

**GATA3**	66 (17%)	0	70 (22%)	1	0.0952
		> 0	248 (78%)	0.599 [0.326–1.1]	

**Ki67**	60 (16%)	< 20	299 (92%)	1	0.000304
		>= 20	25 (8%)	3.58 [1.709–7.488]	

**P53**	55 (14%)	0	272 (83%)	1	< 0001
		> 0	57 (17%)	3.13 [1.745–5.601]	

**ER**	0 (0%)	< 120	153 (40%)	1	0.227
		>= 120	231 (60%)	0.71 [0.406–1.24]	

**PR**	44 (11%)	0	76 (22%)	1	0.941
		> 0	264 (78%)	0.975 [0.499–1.904]	

NE: non evaluable cases on Tissu-micro arrays. **×** Logrank test stratified on chemotherapy.

**Table 8:** Multivariate analysis of ER-positive cases with adjuvant hormonal therapy

**N = 240**		**Coefficient**	**HR**	**IC95**	**p-value**
**VPI**	No		1		
	yes	0.903	2.47	[1.148–5.297]	0.021

**GATA3**	0		1		
	> 0	-0.655	0.519	[0.251–1.076]	0.078

**Ki67**	< 20		1		
	>= 20	1.06	2.9	[1.272–6.604]	0.011

**P53**	0		1		
	> 0	1.08	2.94	[1.505–5.76]	0.0016

## Discussion

The aim of this study was to study the expression of proteins corresponding to genes identified by gene expression profiling to be associated with luminal cases and to determine their impact on the response of patients to hormonal therapy. Due to experimental conditions (e.g. quality of the antibodies), the analysis was limited to 10 proteins. We were able to identify a score combining four factors able to predict the evolution of the luminal cases treated with adjuvant hormonal tamoxifen therapy.

### Molecular subtypes and prognosis

Grossly, our 135 subtyped cases showed a similar distribution of subtypes as found in previous studies [1,2,24]. Our series contained 25% of basal cases, which is within a published range of 17 to 37%. The number of ERBB2 subtype (12%) was slightly higher than in most series. In contrast, the 45% frequency of luminal A was high and the proportion of luminal B was low.

In a previous study [5] we focused on the kinome of luminal A breast cancers. The breast cancer kinome differs between basal and luminal A cases. Within luminal A cases, it allowed the identification of luminal Aa and Ab. Here, we have confirmed the difference in outcome between luminal Aa and Ab by using immunohistochemistry on 43 luminal A cases treated by tamoxifen. The difference between luminal Aa and Ab was due to proliferative factors translated by a higher grade and Ki67 index in luminal Ab than in luminal Aa cases. The fact that a difference could be seen already with a small series suggests the importance of proliferation to distinguish outcome in ER-positive cases whatever their percentage of ER-positive cells.

### Prognosis and hormonal therapy in ER-positive cases

Four factors, VPI, GATA3, P53 and Ki67, were retained by the multivariate analysis.

Two parameters were added in the 9th St Gallen meeting compared with the 8th edition: ERBB2 status and VPI. The volume of data published in the past few years provides compelling evidence for the importance of VPI [25] but the specific impact on luminal cases had never been described. A meta-analysis of microarray data revealed the importance of GATA3 [26]. Its expression in 10-year follow-up [27] demonstrates that its protective effect is more pronounced in patients who received tamoxifen. We showed the prognostic impact of P53 in two previous studies of luminal cases [4,5]. Ki67 higher than 20% is one of the parameters able to distinguish luminal A from luminal B [28] but its specific prognostic impact in luminal cases had not been described.

An important question is whether the combination of VPI, GATA3, P53 and Ki67 predicts pure prognosis or responsiveness to endocrine therapy or both. Few studies using profiling of ER-positive breast cancers treated by tamoxifen have established a signature able to predict the prognosis. The oncotype DX RS [29] is a commercially available assay (Genomic Health, Redwood City, CA) that predicts recurrence in ER-positive cases. It is a PCR-based assay on paraffin-embedded tissue using 16 cancer-related genes and 5 controls. It was validated on 668 node-negative cases in the National Surgical Adjuvant Breast and Bowel Project (NSABP) trial receiving tamoxifen only. The histopronostic grade and recurrence score (RS) were significant. This RS was subsequently validated for chemotherapy and tamoxifen in 645 patients from the NSABP-14 [30]. Only four of the 16 genes are common with the factors we tested here (ER, PR, KI67 and BCL2). A more recent series of 255 ER-positive cases established a signature validated on an independent set of 362 cases coming from different institutions and treated by tamoxifen alone [31]. A total of 181 genes belonging to 13 clusters strongly prognostic (HR = 3.26, *P *= 0.0002). These 13 cluster genes were the most important factor in multivariate analysis.

Immunohistochemistry has been involved in the search for a multiparametric score in ER-positive cases on a series of 257 ER-positive cases treated by tamoxifen; a multimarker model was established from nine markers and five of them were retained in a mathematic model: ER, PR, P53, ERBB2 and MYC. This model was more prognostic than the Nottingham prognostic index [4].

A previous study has looked at oestrogen-regulated genes in the MCF7 breast cancer cell line treated by 17β–oestradiol [32]. These genes were then used to develop an outcome predictor on a training set of 65 luminal breast cancers and then validated on three independent published data sets. Interestingly, two groups of low risk (expressing XBP1, FOXA1 and PR) and high risk (expressing MYBL2 and CCNB2) were distinguished.

The study of a series of 140 cases used 23 antibodies and identified a prognostic score for ER-positive breast cancer without any notion of hormonal therapy [33]. Five factors were retained by Cox analysis (P53, NDRG1, CEACAM5, SLC7A5 and HTF9c) but regression tree analysis retained six factors (P53, PR, Ki67, NAT1, SLC7A5 and HTF9c). The best HR was obtain by the Cox model (HR = 2.21, *P *= 0.0008).

P53 and Ki67 are the two factors common with our series. This again underlines the impact of proliferation in luminal cases. However, our analysis, with four factors, could be an easier manner to study ER-positive cases.

The fact that in our series the patients all received adjuvant tamoxifen stratified on chemotherapy suggests also that these factors could be more than prognostic in cases receiving hormonal therapy.

## Conclusions

Our study of immunohistochemistry factors in luminal breast cancers demonstrates the interest to combine prognostic markers to improve the therapeutic choice.

## Abbreviations

AR: androgen receptor; CI: confidence interval; ER: oestrogen receptor; H&E: haematoxylin and eosin; HR: hazard ratio; MFS: metastasis-free survival; NSABP: National Surgical Adjuvant Breast and Bowel Project; PCR: polymerase chain reaction; PR: progesterone receptor; QS: quick score; RS: recurrence score; TMA: tissue microarrays; TNM: tumour node metastasis; VPI: vascular peritumoural invasion.

## Competing interests

The authors declare that they have no competing interests.

## Authors' contributions

JJ and DB designed the study and wrote the manuscript. JJ and ECJ read all the tissue microarrays in a double-blind manner. FM served as data manager. BE did the statistical analyses. JME, GH and LX contributed to samples and data collection. FB provided DNA microarray data.
